# Epigenetic Mechanisms Involved in the Cardiovascular Toxicity of Anticancer Drugs

**DOI:** 10.3389/fcvm.2021.658900

**Published:** 2021-04-27

**Authors:** Panagiota Papazoglou, Luying Peng, Agapios Sachinidis

**Affiliations:** ^1^School of Pharmacy, Aristotle University of Thessaloniki, Thessaloniki, Greece; ^2^Heart Health Center, Shanghai East Hospital, School of Medicine, Tongji University, Shanghai, China; ^3^Institute of Medical Genetics, Tongji University, Shanghai, China; ^4^Faculty of Medicine, Institute of Neurophysiology, University of Cologne, Cologne, Germany; ^5^Center for Molecular Medicine Cologne, University of Cologne, Cologne, Germany

**Keywords:** induced pluripotent stem cells, hiPSCs, cardiotoxicity, heart failure, genomics biomarkers, anthracyclines, anticancer therapy, epigenetic mechanisms

## Abstract

The cardiovascular toxicity of anticancer drugs promotes the development of cardiovascular diseases. Therefore, cardiovascular toxicity is an important safety issue that must be considered when developing medications and therapeutic applications to treat cancer. Among anticancer drugs, members of the anthracycline family, such as doxorubicin, daunorubicin and mitoxantrone, are known to cause cardiotoxicity and even heart failure. Using human-induced pluripotent stem cell-derived cardiomyocytes in combination with “Omic” technologies, we identified several cardiotoxicity mechanisms and signal transduction pathways. Moreover, these drugs acted as cardiovascular toxicants through a syndrome of mechanisms, including epigenetic ones. Herein, we discuss the main cardiovascular toxicity mechanisms, with an emphasis on those associated with reactive oxygen species and mitochondria that contribute to cardiotoxic epigenetic modifications. We also discuss how to mitigate the cardiotoxic effects of anticancer drugs using available pharmaceutical “weapons.”

**Graphical Abstract:**
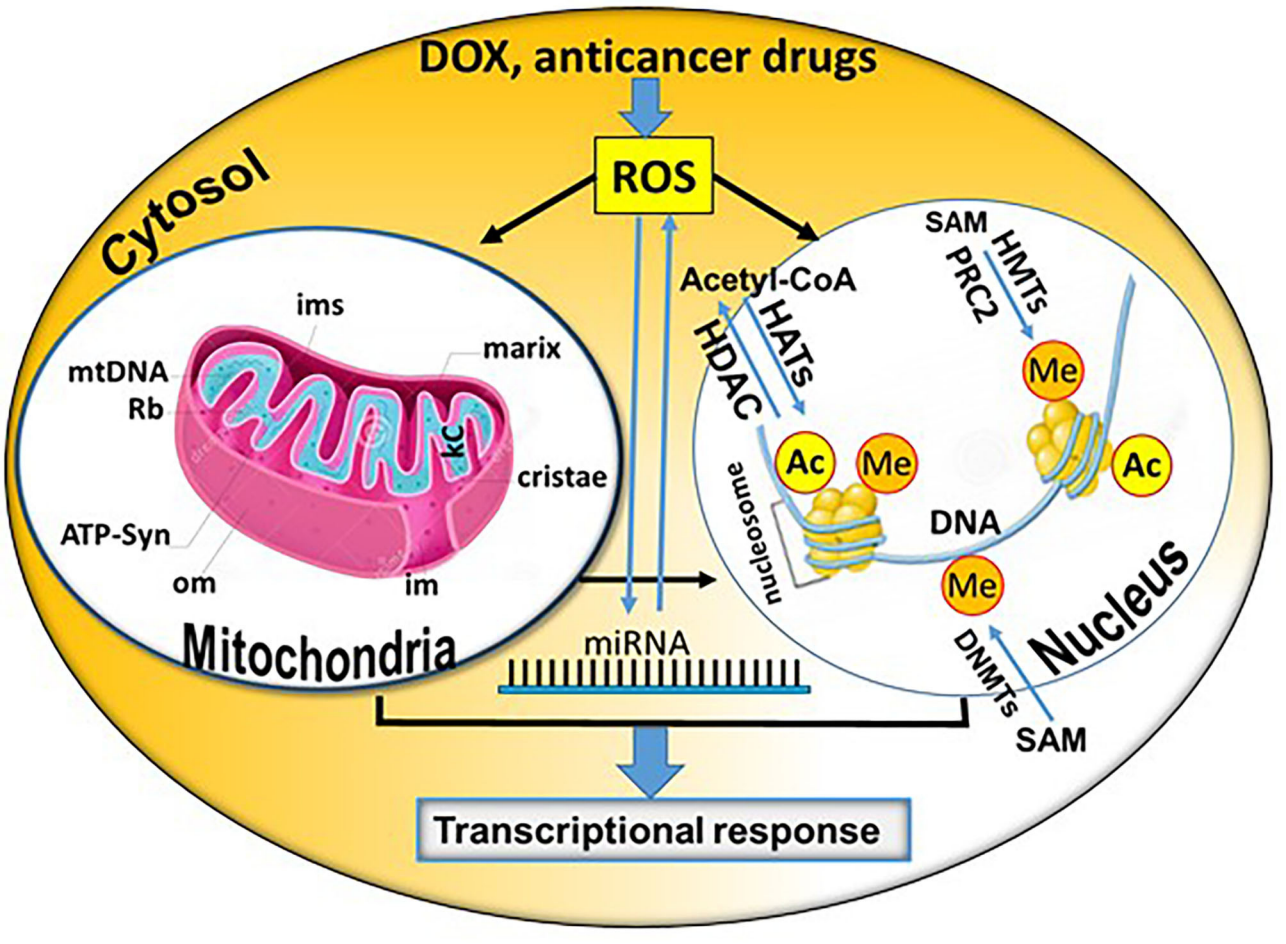
Summary of potential direct and indirect epigenetic mechanisms contributing to the cardiotoxicity of doxorubicin (DOX) and other anticancer drugs (Rb, Ribosomes; kc, Krebs Cycle; om, outer membrane; im, inner membrane; ims, intermembrane space; miRNA, microRNA; SAM, S-adenosyl-L-methionine; Ac, acetylation; Me, methylation; DNMTs, DNA methyltransferases; CoA, coenzyme A; HATs, histone acetyltransferases; HDACs, histone deacetylases; PRC2, RNA polymerase II (RNAPII) can initiate the gene expression process in euchromatin. The polycomb repressive complex 2; HMTs, histone methyltransferases). The core mitochondrion and nucleosome shown in the figure were obtained from Dreamstime.com (https://de.dreamstime.com/) and modified accordingly.

## Introduction

Doxorubicin (DOX; brand name: Adriamycin) was one of the first anthracyclines isolated from *Streptomyces* actinobacterial strains. It is the most commonly prescribed chemotherapy drug to treat breast, ovarian and gastrointestinal cancers, sarcomas, leukemia, non-Hodgkin's and Hodgkin's lymphomas, multiple myeloma and various other cancers, because it shows beneficial therapeutic effects (1). One of its main mechanisms of anticancer activity involves DNA interference, preventing DNA replication and proliferation of the target cancer cells. Moreover, it has been found to interfere, not only with the nuclear DNA, but also with mitochondrial DNA (mtDNA), affecting the energy metabolism (through depletion of adenosine triphosphate; ATP) of several specific cells, including cardiomyocytes (CMs) (2). Another anticancer mechanism of DOX against tumors is the inhibition of topoisomerase IIa (TopIIa), a nuclear enzyme capable of breaking down and reassembling DNA strands in a controlled manner.

DOX acts by producing reactive oxygen species (ROS), causing oxidative stress, damaging cell structures and activating cell death pathways. Patients' responses to this medication are not linear. Indeed, some patients appear to be tolerant to high doses of DOX, while others manifest heart attacks at low-dose (3). ROS play a significant role in the development of cardiovascular diseases (CVDs), including arrhythmia, cardiomyopathy and heart failure (4, 5). Aside from ROS and other peroxide radical-mediated pathways, DOX induces mitochondrial DNA damage and imbalances calcium and/or iron homeostasis (6). One main pathway through antineoplastic drugs is the generation of ROS (7, 8). This process increases oxidative stress, killing cancerous cells, but also generates toxic mediators that affect intact cells by acting on diverse cellular molecules, such as DNA or proteins. Oxidative stress has been implicated as one of the main cellular events related to CM damage. Several factors may be involved in cancer-related cachexia and cardiac impairment. Pro-inflammatory cytokines, such as IL-6, IL-1β and TNF-α, are the main contributors to heart failure. In addition to inflammation, it is worth mentioning that redox regulation is also associated with cancer progression, where elevated levels of ROS are found in most types of tumors (7, 8). Tumors release pro-inflammatory cytokines that can result in oxidative stress, suggesting that an inseparable relationship exists between the production of ROS and an inflammatory status. The tumor itself releases inflammatory cytokines, which are likely important for inducing a ROS niche, favoring new mutations. The C-reactive protein can predict cardiovascular mortality and is commonly used as a biomarker for acute and chronic inflammation (7, 8). In cancer cells, aside from the inhibition of TopIIa activity, DOX also induces cell death, related to redox metabolism. DOX undergoes redox cycling, catalyzed by the cytochrome P540 system. The product of this reaction is the DOX-semiquinone radical (9). This radical causes oxidative damage of tumor cells through the release of iron from its cells. The DOX-iron complexes catalyze oxygen and hydrogen peroxide into potent ROS, which trigger important antitumor responses in cancer cells. Very often, the misbalance between antitumor and adverse effects results in heart injury (9, 10).

Anticancer treatment is not only accompanied by cardiotoxicity, but also vascular toxicity. Common examples of anticancer drugs inducing acute vasospasm are 5-fluorouracil (5-FU) and *per os* administration of capecitabine. These mechanisms suggest activation of the protein kinase C signaling pathway, which leads to imbalanced calcium regulation, causing a dramatic reduction in contractility of vascular smooth muscle cells in the vessels. Arterial vasospasm is manifested by endothelial dysfunction related to the toxic effects of 5-FU on endothelial cells. Patients who have pre-existing endothelial dysfunction, such as those with coronary artery diseases, are at greater risk of developing vasospasm from 5-FU than those who do not. In addition, thymidine phosphorylase, which catalyzes the last step in the conversion of capecitabine to 5-FU, is expressed in atherosclerotic plaques. Therefore, its administration can lead to high local concentrations of 5-FU and increased risk (80%) of developing vasospasm. CVDs that usually occur are angina pectoris (45%), followed by myocardial infarction (22%), arrhythmias (23%), ventricular fibrillation, as well as cases of cardiac arrest and sudden cardiac death (11).

In contrast, paclitaxel therapy has been associated with acute coronary syndrome. Myocardial infarction and myocardial ischemia have been observed up to 14 days after starting paclitaxel therapy. Increased Rho kinase activity in vascular smooth muscle cells of the coronary artery are thought to be associated with angiospasms caused by paclitaxel. Several alkaloids (vinblastine, bleomycin and cisplatin) have been associated with endothelial toxicity that may cause an acute coronary ischemia. These drugs are usually prescribed in combination, although more than 2/3 of patients develop angina pectoris during chemotherapy. Cisplatin can cause acute thrombosis as well as acute vasoconstriction in cancer patients, with severe effects observed even two decades after its administration. In fact, the probability that these patients will develop coronary artery diseases is seven times higher than expected (12).

**Figure 1:**
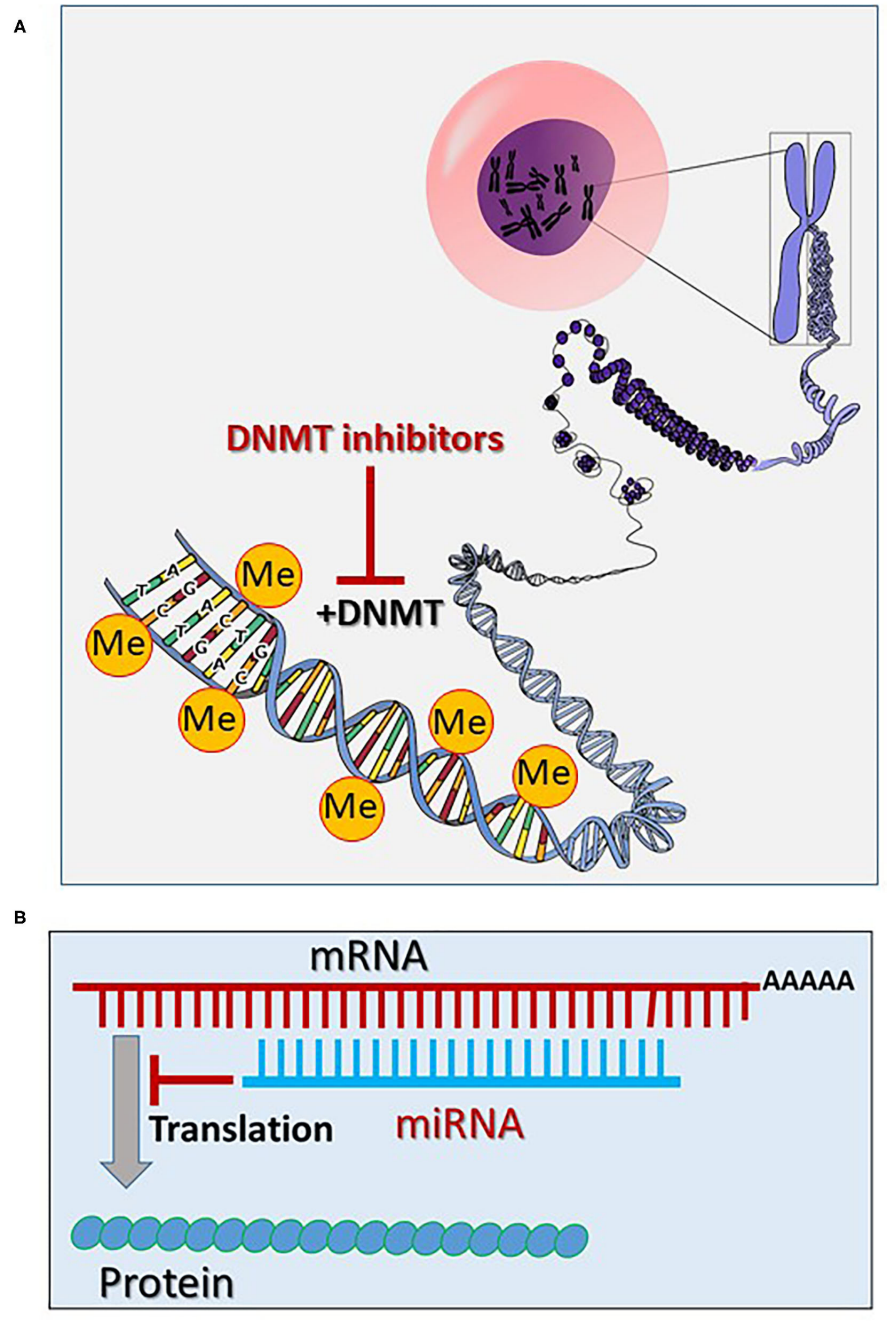
**(A)** Epigenetic mechanisms via methylation of the chromatin DNA (the core figure was obtained from Open Clipart-Vectors; https://pixabay.com/de/users/openclipart-vectors-30363/) and modified accordingly (see section General Aspects of the Epigenetics). **(B)** Inhibition of the mRNA translation via targeting and degradation of the mRNA by microRNAs (miRNAs) (see section General Aspects of the Epigenetics). (Me, methyl; DNMT, DNA methyltransferase).

**Figure 2:**
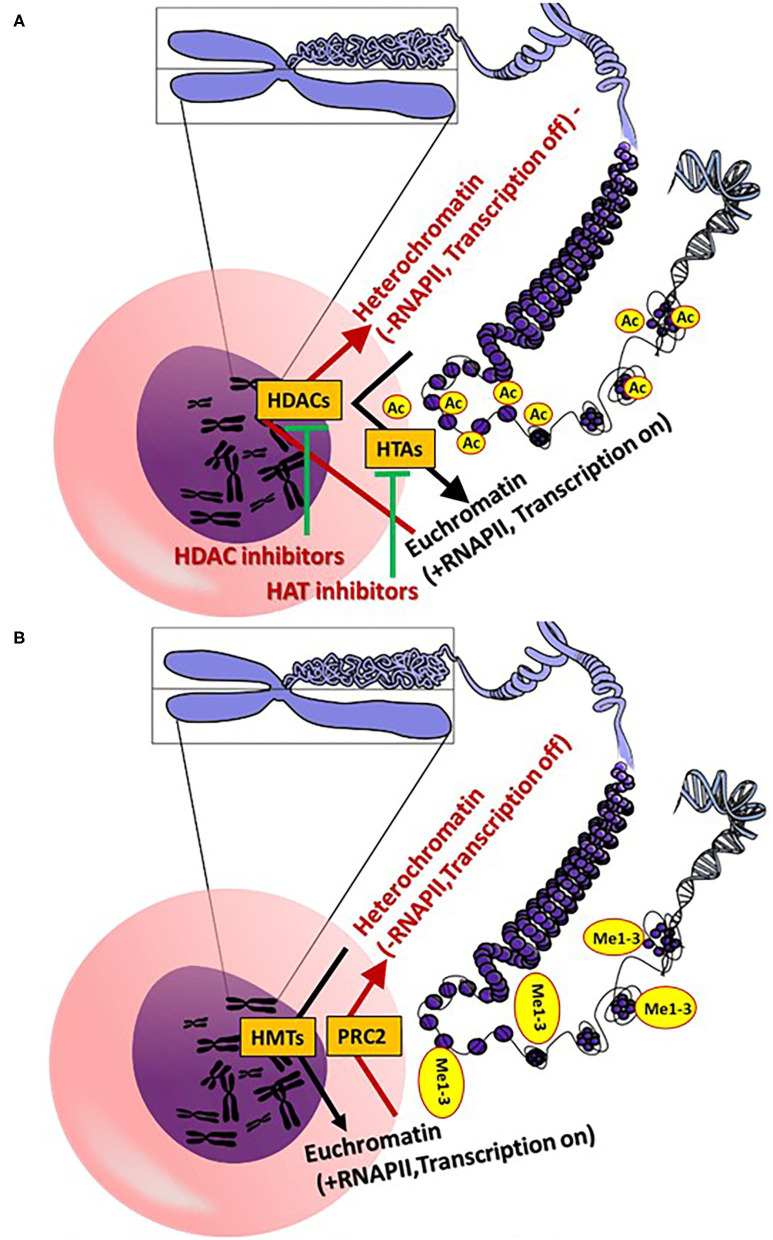
**(A)** General epigenetic action mechanisms of histone acetylation and deacetylation. **(B)** Epigenetic action mechanisms of histone methylation [see section General Aspects of the Epigenetics **(A,B)**: Core figure was obtained from OpenClipart-Vectors (https://pixabay.com/de/users/openclipart-vectors-30363/] and modified accordingly. (Me, methyl; HDAC, histone deacetylases; RNAPII, RNA polymerase II; PRC 2, polycomb repressive complex 2; HMTs, histone methyltransferases; HTAs).

The most important epigenetic mechanisms involve pathways leading to DNA methylation, post-translational histone modifications and regulation of gene expression via non-coded RNAs, such as microRNAs (miRNAs; alternative name miRs) and long non-coding RNAs (lncRNAs) (Figures 1, 2). In addition, epigenetic chromatin alterations occur, linked to environmental factors as well as different drugs that target epigenetic pathways. Epigenetic mechanisms provide transcriptional control in the regulation of gene expression (13). Evidence suggests that epigenetic modifications are associated with changes in both development and behavior, as well as with genetic disorders and diseases. Typically, during the aging of cells, epigenetic changes occur throughout the genome. This process is known as the “epigenetic clock phenomenon” (14). Epigenetic mechanisms not only regulate genomic expression, but also alter drug absorption, metabolism and excretion. The field of pharmacoepigenetics involves study of the variable epigenetic factors that are responsible for differences in patient-to-patient drug responses, new pharmacological targets, as well as disease prognosis and monitoring of long-term biological responses. This scope was expanded, once genetic factors were found to insufficiently explain the differences observed between patients receiving similar or the same treatment regimens (15). Epigenetic mechanisms represent a stable “cell mnemonic” that allows the spread of gene activity from one generation of cells to another. Given that CVDs are responsible for at least 1/3 of premature deaths worldwide, it is worth emphasizing that epigenetic changes are caused by the cardiotoxicity linked to anticancer drugs. How such epigenetic changes can be translated into clinical practice for the development of innovative and effective biomarkers for CVDs remains a challenge (16).

## General Aspects of the Epigenetics

A cell contains ~2 meters of DNA within its nucleus, which is organized into chromatin and further into chromosomes. Each human diploid somatic cell contains 23 pairs of chromosomes, and each chromosome contains several hundreds of thousands of nucleosomes (17). Each nucleosome, which is the core unit of the chromatin, is composed of negatively charged DNA, tightly wound around positively charged histone pairs (H2A, H2B, H3 and H4), forming a histone protein octamer. Epigenetics is the study of processes resulting in gene expression without modification of the DNA sequence; these processes modify the phenotype of an individual without changing the genotype (18). Epigenetic (pathological) modifications can also be triggered by environment/lifestyle, age, disease, chemicals, drugs and toxicants (19, 20).

Epigenetic modifications occur physiologically, mainly via methylation of the chromatin DNA and via methylation and acetylation of chromatin-histones (19, 20). DNA methylation occurs via DNA methyltransferases (DNMTs), which enable the transfer of a methyl group from the S-adenosyl-L-methionine (SAM) to the fifth carbon atom of cytosine bases, forming pairs with guanidine bases (called CpG DNA regions) (Figure 1). Normally, DNA methylation by DNMTs causes a gene expression silencing (21, 22). Demethylation of the CpG DNA regions occurs passively or via the Ten-Eleven Translocation (TET) pathway, increasing gene expression (23).

Histone acetylation of lysine residues (H3K4, H3K9, H3K18, H3K23m H3K27, H3K36-37, H4K8, H4K12, K4H18 H4K20 and H431), as well as on arginine residues (H3R2, H3R8, H3R17, H3R26, H4R3), is catalyzed via the histone acetyltransferase (HAT) enzymes (several isoforms), which induce the transfer of acetyl residues from acetyl-coenzyme A (CoA) to the ε-amino of lysine residues. Deacetylation of a ε-N-acetyl lysine amino acid from histones occurs via histone deacetylases (HDACs) (24, 25) (Figure 2A). Histone methylation is catalyzed by histone methyltransferases (HMTs), with transfer of methyl groups from SAM to lysine and arginine amino acid residues (26, 27) (Figure 2B). Notably, methylation of histones does not alter the charge of the histones, but rather the volume and hydrophobicity of the histones. Alteration of these parameters facilitates binding of effector-specific molecules, forming chromatin-binding complexes. These induce modifications of the chromatin structure (26, 27). Methylation of histone H3 lysine 4 (H3K4me), H3K36me and H3K79me leads to transcriptional activation, whereas methylation of H3K9me, H3K27me3 and H4K20me leads to transcriptional repression via modification of euchromatin (open chromatin; less condensed chromatin) to heterochromatin (highly condensed chromatin) (27). In contrast to heterochromatin, RNA polymerase II (RNAPII) can initiate the gene expression process in euchromatin. The polycomb repressive complex 2 (PRC2; a multi-subunit protein complex) catalyzes tri-methylation of H3K27 to H3K27me3 to regulate the development of multicellular organisms (28). Another key epigenetic regulation of gene expression occurs via histone acetylation and deacetylation of lysine residues (24, 25). In contrast to deacetylation, histone acetylation induces a less tightly packed chromatin (euchromatin), elevating gene expression via RNAPII.

Promising cancer (and cardiovascular) therapeutics that target epigenetics are currently being developed (27, 29, 30). These so-called epi-drugs are small chemical molecules, which act as inhibitors to key epigenetic enzymes, such as DNMTs (e.g., 5-aza-2-deoxycytidine), HATs (curcumin) and HDACs (vorinostat) (29) (Figures 1, 2). Clinical trials with 100 epi-drugs are ongoing, although six epi-drugs have already been approved by the US Food and Drug Administration (FDA) (27) (Table 1). Other epigenetic modulators are miRNAs, which regulate gene expression without modifying the gene sequence. They act via specific targeting of mRNAs using complementary sequences, leading to degradation of the appropriate mRNA, which affects protein levels (Figure 1B). In addition, the expression of miRNAs can also be regulated by epigenetic mechanisms, including DNA methylation and histone methylation and/or histone acetylation (31). Several miRNA-targeted therapeutics have reached clinical phase trials for treating of cancer and hepatic diseases (32).

**Table 1:** Epi-drugs approved by the US Food and Drug Administration against cancer and bipolar disorders (27).

**Therapeutic compound/drug name**	**Therapeutic target**	**Epidrug class/biological effect**	**Interventional clinical trials status**	**Number of studies**	**Same pathologies/disorder investigated**
Azacitidine (5-Azacytidine)/Vidaza	DNMT1	DNA Methylation Inhibitor	FDA-approved		Myelodysplastic Syndrome
Decitabine (5-aza-2'-deoxycytidine)/Dacogen	DNMT1	DNA Methylation Inhibitor	FDA-approved		Myelodysplastic Syndrome
Belinostat/Beleodaq	HDACs	Histone Deacetylation Inhibitor	FDA-approved		Relapsed or refractory peripheral T-cell Lymphoma
Romidepsin/Istodax	HDACs	Histone Deacetylation Inhibitor	FDA-approved		Cutaneous T-cell Lymphoma.
Panobinostat (Hydroxamic Acid)/Farydak	HDACs	Histone Deacetylation Inhibitor	FDA-approved		Multiple Myeloma
Valproic acid/depakene and Stavzor	HDACs	Histone Deacetylation Inhibitor	FDA-approved		Bipolar disorder, Adjunctive Therapy in Multiple Seizure

*HDACs: Histone deacetylases; DNMT: DNA methyltransferase*.

## Direct and Indirect Epigenetic Dysfunctions Caused by Doxorubicin

Manifestations of DOX's cardio toxicity include the development of fibrotic lesions, disordering of the CMs and significant deregulation of the transcription processes. Interestingly, even at low doses of DOX, changes occur to the transcriptional profile of many HDACs, which are known epigenetic regulators of cardiac configuration (33). Therefore, a novel cardio protective therapy, based on targeting HDAC enzyme activity during DOX therapy, remains an attractive goal (34). Pathological processes leading to heart dysfunction and heart failure are caused by a cascade of rapid post-translational modifications, governed by a strong epigenetic mechanism. This is most likely caused by HDACs, which play a key role in histone or protein deacetylation, and consequently, in controlling total gene expression (12). When it comes to anticancer treatment with DOX, there are many examples of how epigenetic aspects affect the response of cancer cells to drugs. For example, estrogen enhances the sensitivity of breast cancer cells to both DOX and cisplatin via a self-induced hypermethylation mechanism. More recently, it has been reported that mutations in SETD2, a trimethyltransferase-3-lysine-36 (H3K36me3), have led to increased resistance to chemotherapy through *in vitro* and *in vivo* leukemia models. In terms of cardiotoxicity, there has been a growing interest in sirtuin proteins (SIRTs), a category of NAD^+^-dependent deacetylases that catalyze the deacetylation of many proteins, including histones. These proteins have been shown to play an important role in cardioprotection against the cardiotoxicity induced by DOX (35). ROS has been discussed as a key modifier of the epigenetic landscape, mediating the adverse effects of ROS on cancer and CVDs (36, 37). In this context, ROS affect DNA methylation, histone modifications (acetylation and methylation) and non-coding RNA expression [extensively reviewed by (36)]. Consequently, changes in the epigenetic landscape result in abnormal gene expression in both the nucleus and mitochondria, mediating development of CVDs.

Disruption of normal mitochondrial function has been shown to be one of the leading causes of heart injury, consistent with the fact that mitochondria are responsible for producing about 90% of the ATP metabolized by CMs. It is well-known that, in addition to its high affinity for DNA, DOX and other anthracyclines interact with cardiolipine, a mitochondrial negatively charged phospholipid. The interaction of DOX with cardiolipine causes a reversal of the electron transport chain, because cardiolipine is required for normal electron transport chain function and activity, as well as healthy functioning of the cell's respiratory chain (38). Changes in DNA methylation levels are particularly evident in the kelch-like family member 29 (Klhl29) and the Nicotinamide mononucleotide adenylyltransferase 2 (Nmnat2) gene, where they are associated with changes in mRNA gene expression. It is important to note that DOX may lose an electron catalyzed by the dehydrogenation of mitochondrial NADH, causing formation of free radicals. Oxidative stress, in turn, damages proteins, DNA and membranes, and is also involved in the induction of mitochondrial permeability. The increased permeability of the internal mitochondrial membrane leads to depolarization and deregulation of mitochondrial energy production (38). The epigenetic remodeling caused by DOX may be responsible for disrupting mitochondrial energy metabolism, but the opposite may also be valid, because the formation of most of the metabolites needed for epigenetic chromatin changes is associated with mitochondrial pathways that are affected by redox cell reactions. DOX can suppress the expression of genes required for oxidation of beta fatty acids and ATP-producing mitochondrial enzymes, explaining the reduced formation of epigenetic modifiers of mitochondrial metabolites, such as acetyl-CoA, acetylcarnitine and ATP (3). A different pattern of protein acetylation has been found in cardiac mitochondrial fractions of rats receiving DOX, accompanied by increased activity of HDACs, suggesting a mutation-predisposition between mitochondrial dysfunction and epigenetic alterations connected with DOX-induced cardio toxicity. Finally, the selective toxicity of DOX in cardiac mitochondria, in contrast to the liver, may be related to the rate of renewal of the mitochondrial cycle. In the heart, this renewal cycle is about 14 days, while in the liver it is only 2–4 days. Thus, liver mitochondria recover faster after DOX toxicity. Mitochondria also act as a source of metabolites and other factors, which are epigenetic modifiers. One of the main epigenetic modifiers is SAM, which is synthesized from ATP (produced in mitochondria) and methionine. The reaction is catalyzed by the enzyme methionine adenosyl transferase (MAT) (39). SAM serves as a general substrate for DNA and histone methylation. Thus, it is believed that mitochondrial disruption induced by DOX and other anticancer drugs may be the main cause for epigenetic changes in the CMs' chromatin (40, 41). The mitochondrial damage caused by DOX is cumulative and persistent, similar to that observed clinically for congestive heart failure (42). Recently, the so-called “mitochondrial memory” or the irreversible nature of DOX in mitochondrial toxicity has been studied in animals (42). When DOX (2 mg/kg) injections were performed weekly, they resulted in cumulative, dose-dependent increases in concentrations of 8-hydroxyguanosine, both in nuclear (nDNA) and mtDNA (34). DOX concentrations were 50% higher in cardiac mtDNA than in liver mtDNA, and remained elevated for 4 weeks after the last DOX injection (34). Therefore, DOX appears to be selectively detrimental to the heart. DOX interrupted cardiac mitochondrial biogenesis, and reduced mtDNA levels as well as variable cross-sectional transcripts for multiple mitochondrial genes encoded by both nuclear and mitochondrial genomes. The transcription of genes involved in lipid metabolism and epigenetic formation were also affected. Transcription of mtDNA is paramount to functional mitochondrial biogenesis. Thus, quantification of the transcription levels of genes involved in this process can be used as a parameter for prediction of abnormal mitochondrial functions. One of the main predictive genes is the peroxisome proliferator-activated receptor γ coactivator 1-alpha (PGC-1α), which is the main regulator of mitochondrial biogenesis. Typically, its meta-transcript levels decrease when abnormal mitochondrial functions occur (42). Likewise, the transcript encoding for the mitochondrial transcription factor A (TFAM), which controls mtDNA transcription, integrity and copying, also is a sensitive indicator of abnormal mitochondrial function. It was also found to be reduced (3). Because of DOX-induced reduction of TFAM, a reduction in the quality and quantity of mtDNA occurred. Typically, DOX reduces mtDNA by 50%. Furthermore, there is a selective reduction in the enzymatic activity of cytochrome oxidase (COX; a complex of multiple subunits, encoded by both nDNA and mtDNA) vs. the activity of electrical dehydrogenase (SDH) encoded entirely by nDNA. This again points to a mitochondrial transfer imbalance (43, 44). A reduction in ATP synthesis would be expected because of such imbalance. Indeed, ATP synthesis in hiPSCs-derived CMs is significantly reduced after cell treatment with etoposide and DOX (43, 45, 46). As reduction of mitochondria biogenesis and mitochondria functionality is severely affected by DOX, in turn, the mitochondria-dependent synthesis of SAM will be significantly reduced. We expect that epigenetic modifications of DNA and histones also will be imbalanced.

Excessive ROS production affects the expression of miRNAs and *vice versa* (5, 47, 48). In this context, inhibition of miR-25 in animal CMs exerted beneficial effects on the DOX-induced apoptosis, ROS production and DNA damage related to targeting of the phosphatase and tensin homolog deleted on chromosome 10 (PTEN) (49). In general, changes in miRNA expression mainly occur via the Nrf2, calcineurin/nuclear factor of activated T cell (NFAT), or via the nuclear factor kappa B (NF-κB) pathways (5). ROS-induced heart injury clearly affects the expression of many miRNAs. To date, several circulating miRNAs (miRNA-499, miRNA-199, miRNA-21, miRNA-144, miRNA-208a, miRNA-34a) have been reported to be potential biomarkers of ROS-associated CVDs (5).

Cardiovascular toxicity is manifested by elevated levels of the classical biomarkers in the blood, such as cardiac troponin I (cTnI) and cardiac troponin T (cTnT). These elevated levels are well-correlated with myocardial injury and act as important plasma biomarkers for the diagnosis of cardiac damage in clinical and preclinical studies. However, high levels of these biomarkers occur only after heart damage. Unfortunately, these biomarkers can only be detected a few hours after myocardial infarction and after treatment with cardiotoxic drugs. Therefore, identification of novel pharmacogenetic early biomarkers capable of predicting cardiac damage are urgently needed. For example, altered expression of miRNAs was recently found *in vivo* and *in vitro* in a study to develop early biomarkers for DOX cardiotoxicity. One of the most important epigenetic mechanisms is the gene regulation induced by the miRNAs and lncRNAs. In this context, after exposure of mice to different cumulative doses, pre-apoptotic miR-34a was detected in large concentrations, indicative of the first stages of myocardial alteration (50).

For the development of more human-related toxicity testing systems *in vitro*, human embryonic stem cells and human-induced pluripotent stem cells (hiPSCs) have been used to predict the adverse effects of different compounds on genome and epigenome levels (51). Recently, genomic biomarkers have been identified after brief treatment of CMs derived from hiPSCs (hiPSC-CMs) with different anticancer drugs, such as anthracyclines and etoposide (43, 46, 52, 53). Gene ontology analysis of the differential gene expression indicates that processes regulating the contraction of CMs were inhibited by several anticancer drugs, whereas processes promoting apoptosis of the CMs (stress response, pathway p53 signaling) were up regulated (43, 45, 46, 52, 53). In addition, 14 miRNAs were identified as genomic biomarkers for cardiotoxicity after treatment of human CMs with DOX (52) and etoposide (43). Interestingly, among the genomic cardiotoxicity biomarkers identified under *in vitro* conditions, several were also identified in patients suffering heart failure (53). Furthermore, metabolite signatures of DOX-induced toxicity in hiPSC-CMs have been identified. Repeated exposure of human CMs to DOX caused reductions in the use of pyruvate and acetate, and accumulation of formate in the culture medium (54).

## Prevention of Cardio Toxicity Induced by Anticancer Therapy

Several compounds mitigate anticancer drug-induced cardio toxicity. Rutin (a poly phenolic flavonoid) may be a protective agent against cardio toxicity of anticancer drugs via antioxidant and anti-inflammatory mechanisms. Analysis of the hearts of mice that had undergone cardio toxicity from DOX included inhibition of overactive autophagy and apoptosis mediated by Ak strain transforming activation. Rutin caused a reduction in cardiac fibrosis and morphological changes in the heart linked to treatment with DOX (55). Resveratrol, another poly phenolic flavonoid, has been shown to reduce cardio toxicity linked to DOX by increasing mitochondrial biogenesis. This in turn stimulates the heme oxygenase (HO)/CO system, which acts as cytoprotective in several different tissues and cell types (56, 57). Dexrazoxane is the only FDA-approved protective treatment for DOX cardio toxicity. Its mechanism of cardio protection involves an indirect antioxidant effect via formation of iron complexes. Inhibition of cardiac topoisomerase II-β, as well as the inhibition of DNA cleavages caused by DOX, also are considered cardio protective mechanisms. Likewise, carvedilol, a non-selective β -blocker, appears to improve cardiac mitochondrial function, resulting in increased calcium-loading capacity during DOX therapy (33). Finally, studies have shown that HDAC inhibitors are effective in reducing cardiac hypertrophy under pathological conditions and in weakening the structural remodeling after myocardial infarction. A detailed map of chromatin modification-induced by two HDAC inhibitors, chostatin A (TSA) and suberoylanilide hydroxamic acid (SAHA), has been described in a model of human aortic endothelial cells. HDAC inhibitors mitigate the cytotoxic effect of DOX on the heart. Repeated animal studies showed that treatment of patients with SAHA (an inhibitor of class I and II HDACs) led to a significant improvement in cardiac function (58). The novel butyrate derivative phenylalanine-butyramide protects from doxorubicin-induced cardio toxicity in mice probably via involving HDAC associated mechanisms (59). Till know detail epigenetic mechanisms involved in cardio toxicity were reported by doxorubicin and other anthracyclines only on miRNA level (52).

There are some evidence that female animal hearts are protected due to 17-β-estradiol from the doxorubicin-induced cardio toxicity through the increased levels of ROS and apoptosis in male hearts. However, potential clinical cardio-protective benefits of females hearts are controversial discussed (60).

## Conclusions

Undoubtedly, anticancer treatments cause a persistent and long-term cardiovascular toxicity, leading to CVDs. Thus, an understanding of the toxicity pathways induced by anticancer therapeutics is a priority and precondition to developing safer and better therapeutic drugs for treatment and to ensure a better quality of life for cancer patients. In the last decade, it was recognized that targeting of epigenetic regulators, such as HDACs, prevents severe cardiovascular toxicity during anticancer treatment. Therefore, promising efforts are ongoing to develop epigenetic drugs, targeting HDACs, to prevent the severe side effects of the anticancer drugs. Classical biomarkers, such as elevated concentrations of troponins in the blood, can only be detected after myocardial infarction and cardio toxicity. Human-relevant test systems based on pluripotent stem cell-derived CMs significantly contributed to the identification of several key cardio toxicity mechanisms, involving both genetic and epigenetic pathways. These studies also lead to identification of genetic and epigenetic markers, such as miRNAs, which may be applied as early biomarkers for predicting and preventing cardiac damage induced by anticancer treatment. Mitochondrial dysfunctions in CMs induced by the anticancer drugs via classical and epigenetic pathways are key cardio toxicity mechanisms. Furthermore, epigenetic remodeling, mediated by anticancer drugs, may disrupt the mitochondrial energy metabolism. We conclude that several genetic and epigenetic targets of anticancer therapeutics have now been identified for preventing or ameliorating cardiovascular toxicity. However, clinical studies still need to confirm the beneficial nature of these so-called epi-drugs.

## Author Contributions

PP, LP, and AS drafted and wrote the manuscript. All authors contributed to the article and approved the submitted version.

## Conflict of Interest

The authors declare that the research was conducted in the absence of any commercial or financial relationships that could be construed as a potential conflict of interest.
